# Population Structure and Gene Flow of the Yellow Anaconda (*Eunectes notaeus*) in Northern Argentina

**DOI:** 10.1371/journal.pone.0037473

**Published:** 2012-05-24

**Authors:** Evan McCartney-Melstad, Tomás Waller, Patricio A. Micucci, Mariano Barros, Juan Draque, George Amato, Martin Mendez

**Affiliations:** 1 Department of Ecology, Evolution and Environmental Biology, Columbia University, New York, New York, United States of America; 2 Sackler Institute for Comparative Genomics, American Museum of Natural History, New York, New York, United States of America; 3 Fundación Biodiversidad – Argentina, Buenos Aires, Argentina; 4 Wildlife Conservation Society, Bronx, New York, United States of America; University of Lausanne, Switzerland

## Abstract

Yellow anacondas (*Eunectes notaeus*) are large, semiaquatic boid snakes found in wetland systems in South America. These snakes are commercially harvested under a sustainable management plan in Argentina, so information regarding population structuring can be helpful for determination of management units. We evaluated genetic structure and migration using partial sequences from the mitochondrial control region and mitochondrial genes cyt-*b* and ND4 for 183 samples collected within northern Argentina. A group of landscape features and environmental variables including several treatments of temperature and precipitation were explored as potential drivers of observed genetic patterns. We found significant population structure between most putative population comparisons and bidirectional but asymmetric migration in several cases. The configuration of rivers and wetlands was found to be significantly associated with yellow anaconda population structure (IBD), and important for gene flow, although genetic distances were not significantly correlated with the environmental variables used here. More in-depth analyses of environmental data may be needed to fully understand the importance of environmental conditions on population structure and migration. These analyses indicate that our putative populations are demographically distinct and should be treated as such in Argentina's management plan for the harvesting of yellow anacondas.

## Introduction

Genetic data offer high resolution and power for evaluating population structure and dispersal patterns, which is especially useful in species that are difficult to find or observe such as yellow anacondas. Combined with landscape information, genetic approaches can increase our understanding of spatial, environmental and even ecological constraints to dispersal. Yellow anacondas in northern Argentina are good candidates for these types of landscape genetics studies as they are found in a heterogeneous environment, with presumably limited opportunities for dispersal between populations [1]. They require wet, swampy habitats, and as such can mainly disperse along rivers and floodplains and their associated vegetative habitats [1], [2].

Several mechanisms have been proposed for the formation of spatial genetic structuring; these mechanisms may act individually as main drivers or act in concert. Gene flow between populations might simply be limited due to the physical distance between groups, creating a spatial genetic pattern known as isolation by distance (IBD) [3]. Instead or in addition to IBD, environmental variables such as temperature, precipitation, etc. may be important in limiting dispersal, a phenomenon known as isolation by environmental distance (IBED) [4]. Furthermore, landscape features such as presence and directionality of rivers (both present and historic) can contribute to our understanding of relationships between populations [5]–[8]. By jointly evaluating the spatial patterns of genetic structure and magnitude and directionality of gene flow between yellow anaconda populations in this heterogeneous area, we can better understand factors influencing dispersal in these and possibly other large semiaquatic snakes.

*Eunectes notaeus* is a commercially-important species that was heavily exploited for their valuable skins until the late 1990s [2]. Additionally, manmade disturbances such as deforestation, wetlands drainage and heavy damming of the Paraná River are disrupting natural hydrological and alluvial patterns, leading to an irregular tempo and intensity of flooding with unpredictable effects on anaconda populations [9]. In 2002, a sustainable harvest plan for yellow anacondas was initiated in the province of Formosa, Argentina, to reconcile the traditional hunting of this species with its long term conservation [2], [10]. In this context, evaluating for population structure in northern Argentina is important for identifying potential management units and priority areas for conservation [11]–[13].

A previous study by Mendez et al. [1] found preliminary evidence of population structure between groups of yellow anacondas in northern Argentina, suggesting dispersal constrained to habitat along rivers. This study, however, was conducted with relatively small sample sizes and only two genetic markers (ND4 and cyt*-b),* with a resulting low degree of resolution. The current study aims to carry out a more detailed evaluation of population structure and connectivity in relation to presumably relevant habitat features, as well as estimating effective migration rates between anaconda groups in northern Argentina. Understanding the connections and movement between these populations will increase our knowledge about the species'ecology and demography and some of the environmental or ecological drivers of population structuring. This, in turn, will be helpful for the sustainable harvesting and management of yellow anacondas in Argentina [2].

## Materials and Methods

### Habitat description

**Figure 1:**
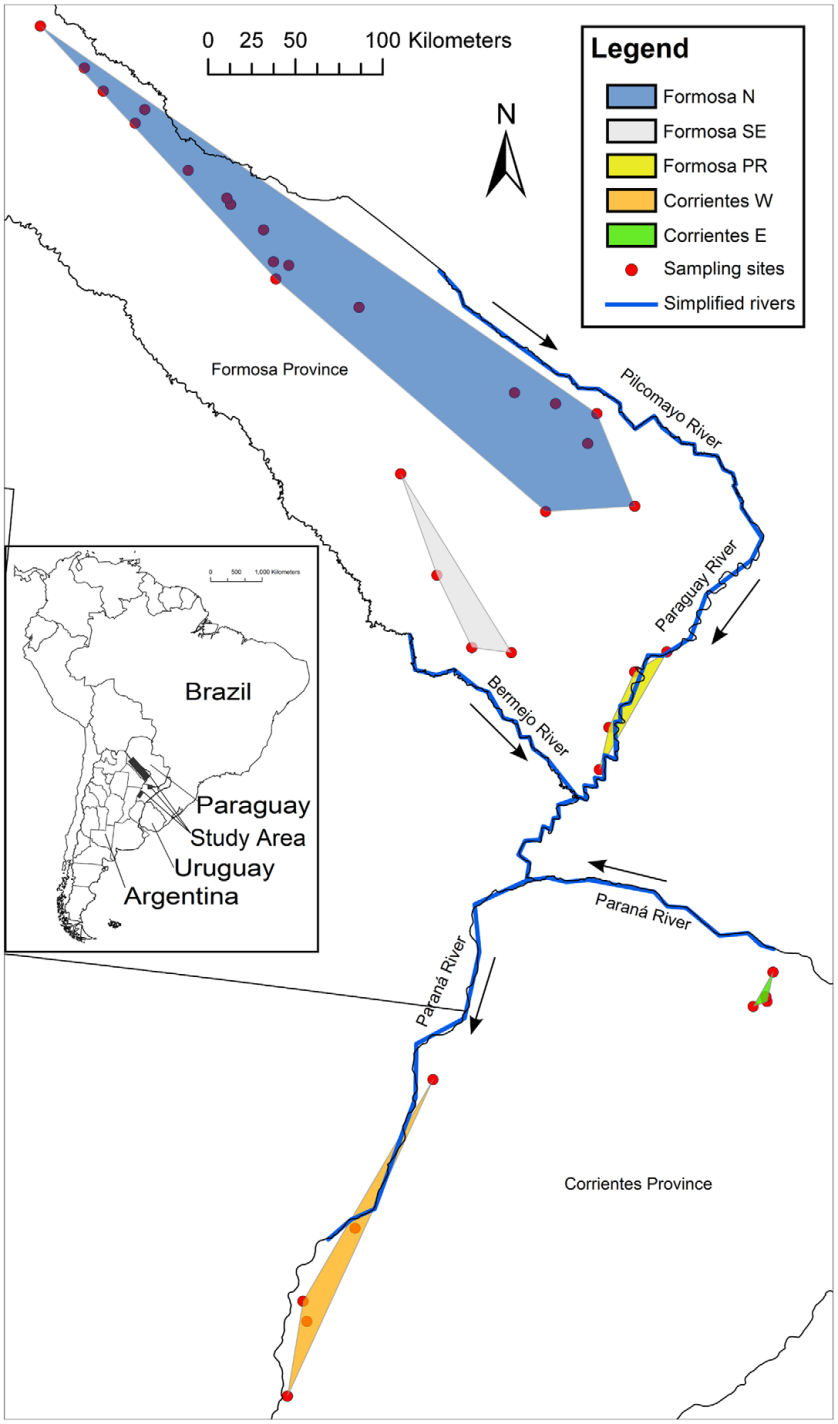
Map of study area and putative populations. Study area and distribution of sampling sites with putative population assignments shown as colored polygons. Arrows represent directionality of flow of adjacent river. Map projection: UTM Zone 21S, WGS 1984, Central Meridian: −57.000, Latitude of Origin: 0.000.

Yellow anacondas occur from the Pantanal region in Brazil and Bolivia, throughout Paraguay, to northeastern Argentina. Our study area encompasses the Argentinean portion of the species range, in the Formosa and Corrientes provinces (Figure 1). This region represents the southernmost part of the range of *Eunectes notaeus*, extending as far down as 30° S [10], [14]. Most of the area is a poorly-drained flat plain where palm savannas, grasslands and forest patches form a matrix of wetlands and creeks that slowly drains into four major rivers: the Pilcomayo, Bermejo, Paraná, and Paraguay. Both the Pilcomayo and Bermejo rivers flow to the southeast, while the Paraguay flows to the south. The Paraná River flows west and forms a border between Argentina and Paraguay until it is joined by the Paraguay River, where it turns south (Figure 1).

In order to evaluate the genetic structure of yellow anacondas in our study area, we grouped sampling sites into five putative populations based on environmental factors. In defining our populations we considered extensive dry areas as barriers to dispersal of *Eunectes notaeus* and continuous wetland systems as areas where gene flow is not prevented other than by geographical distance. We also considered the different habitat types and wetland systems as possible isolating factors, as described below. The defined putative populations are Formosa N, Formosa PR, Formosa SE, Corrientes E and Corrientes W (Figure 1).

Highly suitable habitats for *Eunectes notaeus* exist in Formosa Province. Most of them are the palm and wetland savannas of the Humid Chaco ecoregion [2], [15], the prevalent habitat types for populations Formosa SE and Formosa N. The putative population Formosa PR is found on the eastern limit of Formosa Province on the Paraguay River, an island and delta type ecosystem that is characterized by an extensive floodplain covered by riparian forests and oxbow lagoons [10], [15]. To the west, the Pilcomayo River has been regressing into the Dry Chaco ecoregion [15] over the past several decades, forming a 3,000 km^2^ floodplain known as the Bañado la Estrella [10], [16], which is located within population Formosa N. This highly seasonal marsh is characterized by the presence of palms mixed with dead forest patches covered by climbing vegetation, and is flooded during the local summer, after which it progressively dries out until ninety percent of the land is again visible [10].

The Paraguay River floodplain continues to the south into Corrientes province along the Paraná River and adjacent wetlands [15] but with average temperatures progressively descending along the latitudinal gradient. The putative population Corrientes W is found within these wetlands surrounding the Paraná River. In the interior sectors of the province of Corrientes there are several characteristic swamp systems that are located along the ancient beds (alluvial cone) of the Paraná River, before this river adopted its current position. These swamps, which contain the putative population Corrientes E, are locally known as Iberá ecoregion [15], and are less seasonal and limnologically different with regard to most of the Humid Chaco and Paraná-Paraguay river wetlands.

Multiple possible mechanisms of isolation were considered in the assignment of our five putative populations. Generally, Formosa SE and Corrientes E are not strongly connected to the large river systems but are found in relatively isolated wetland systems that behave independently and are modulated by local rains. Within the riverine populations, Formosa N is an interconnected system of wetlands strongly influenced by the Pilcomayo River running down from the Andes. Formosa PR is closely associated with the Paraguay River that is mainly modulated by the Pantanal in Brazil, while Corrientes W receives the effects of both the Paraguay and Parana rivers. The difference in timing of flooding between these rivers may lead to temporal isolation of these habitat areas. Finally, Corrientes W occurs far downstream of the other populations at the southern edge of the species range, where significant stretches of unsuitable habitat between populations are expected to occur.

Yellow anacondas are abundant in these areas of northern Argentina, and are most easily found during winter when they emerge from water to bask [10]. Although elevation does not seem to be an important factor in the study area, as the entire region lies below 200 meters above sea level, the presence of dry sectors between wetlands are expected to significantly affect dispersal and gene flow in this semiaquatic species. However, short seasonal movements of a few hundred meters over dry areas between adjacent wetlands are common, particularly during the dry season (T. Waller, personal observation).

### DNA extraction and amplification

Blood samples were obtained from 183 yellow anacondas from 36 sampling sites within Formosa Province and Corrientes Province (Fig 1) and exported under CITES permit numbers 22484, 22485, and 35566. Genomic DNA was extracted using the QIAamp Blood and Tissue kit (Qiagen, Hilden, Germany). Partial sequences of the mitochondrial genes cyt-*b* and ND4 were amplified and sequenced using primers and methods as described in Mendez et al. [1]. Because the mitochondrial control region has been duplicated in *Eunectes notaeus* [17], we designed primers to target and amplify only one of these regions for our analysis. Primers were designed with the forward primer (ENCR1F: GGTCCCCAAAACCAGAATTT) located 54 base pairs (bp) upstream of the control region within tRNA-proline, and the reverse primer (ENCR1R: AGGGGCTCCACCTTGACTA) 691 bp downstream within the control region. A single control region was then amplified using the following thermal profile: preliminary denaturation for 3 minutes at 94°C followed by 40 amplification cycles consisting of 30 seconds of denaturation at 94°C, 1 minute of annealing at 56°C, and one minute of elongation at 72°C, with a final extension period of 5 minutes at 72°C. Sequencing was carried out on an ABI 3730×l using Big Dye terminator (Applied Biosystems, Foster City, CA).

### Data analysis

Sequences were aligned using ClustalW in MEGA 5.03 with a gap opening penalty of 15, a gap extension penalty of 6.66, and a transition weight of 0.5 [18], [19] and concatenated using Sequence Matrix 1.7.8 [20]. DNAsp 5.10.01 [21] was used to define haplotypes and also to evaluate genetic diversity by calculating haplotype diversity and nucleotide diversity [22] of the fully-concatenated sequences.

We first visualized the overall structure of the genetic data and potential spatial patterns of genetic diversity through the construction of haplotype networks. We used networks to visualize such relationships as they are more appropriate than trees at depicting data in which ancestral haplotypes are still present [23], [24]. Median-joining networks [25] were created using the software Network 4.6.0.0 (www.fluxusengineering.com).

We evaluated genetic structuring between our putative populations by computing the pairwise fixation indices Fst (using haplotype frequencies) [26] and Φ_st_ (using pairwise differences between haplotypes) [27] in Arlequin v3.5.1.2 [28]. Fixation indices were tested for significance using 10,000 permutations of the data. We further evaluated structure using the exact test of population differentiation [29], [30] in Arlequin with one million steps in the Markov chain and 100,000 dememorization steps. We did not apply a correction for multiple tests to significance levels [31], [32].

We were particularly interested in the potential mechanisms that may cause the observed genetic structure and gene flow. As a first approach to this question, we evaluated the importance of a suite of spatial and environmental variables to the observed genetic patterns. We evaluated the plausibility of a pattern of IBD for the arrangement of populations in our study using a regression of standardized fixation index (i.e. F_st_/(1–F_st_) and Φ_st_/(1–Φ_st_)) on geographic distance [33]. First, polygons were drawn to represent putative populations by connecting the fewest number of sampling sites that bounded all sites within the populations, and centroids of the polygons calculated in ArcMap 9.3.1 (ESRI). Geographic distances between populations were first calculated as straight-line distance between centroids. Alternatively, along-river distance was calculated as the shortest distance from centroid to a major river, and following a simplified river path to the next centroid. For tests of IBD, straight-line distance was log transformed while along-river distance was treated as a linear habitat and untransformed, as suggested by Rousset [33]. Regression analyses and Mantel tests [34] were performed using 100,000 randomizations of the data in the program Isolation by Distance 1.52 [35]. We also evaluated the plausibility of patterns of IBED, where some environmental variables would better explain the genetic distance patterns [4]. Worldclim data (1 km resolution) [36] was used to represent the following suite of relevant climatic variables: average monthly precipitation, driest month average precipitation, whole-year mean temperature, coldest-month mean temperature, and coldest three months mean minimum temperature. These environmental variables were tested for correlation to genetic distances between populations while controlling for the effect of spatial distance by conducting partial Mantel tests [37] with 100,000 randomizations in Isolation by Distance 1.52.

To complement this approach, we evaluated the possibility of asymmetric gene flow in the study area, as this information may enhance our understanding of the relative roles of the rivers and associated areas in mediating gene flow for this species. We estimated asymmetric migration rates using the maximum likelihood procedures implemented in the software MIGRATE [38]. MIGRATE provides estimates of M (m/µ) and *θ* (2N_e_µ) where m is the immigration rate, µ the mutation rate, and N_e_ the effective population size. The product *θ* M results in the number of immigrants per generation 2N_e_m. We adopted a migration matrix model allowing for asymmetric migration rates between populations and variable subpopulation sizes. Our migration model prevented gene flow between populations Formosa N and Corrientes E or Corrientes W, since Formosa PR is a stepping-stone between them. We ran five replicates of a Markov chain scheme to produce initial values for our parameter estimation. Here, our data was tested with default starting values for the population size and M parameters, in 5 independent runs of the Markov chain scheme: 20 short chains (dememorization: 10,000 genealogies, recorded genealogies: 2500, sampling increment: 100), and 3 long chains (dememorization: 10,000 genealogies, recorded genealogies: 25,000, sampling increment: 100). Using as initial parameters the consistent resulting values from these five initial runs, we launched three series of longer Markov chain schemes to estimate our parameters of interest. In the first series (s1), we launched in parallel 10 runs with 10 independent replicates each of the following Markov chain scheme: 15 short chains (dememorization: 10,000 genealogies, recorded genealogies: 2500, sampling increment: 100), and 5 long chains (dememorization: 10,000 genealogies, recorded genealogies: 25,000, sampling increment: 100). The second series (s2) was a run consisting of 100 independent replicates of the same Markov chain scheme and starting parameter set. The third series (s3) was another run with 100 replicates of the same Markov chain scheme and increased starting M values (all initial M values multiplied by 100), to ensure a wider exploration of the parameter space.

For the first series we report the average results of the 10 individual runs and the frequency of runs that resulted in non-zero M values, to illustrate the relative importance of individual runs. For the second and third series we simply report the resulting final matrices, each with the likelihood-weighted mean pairwise population size and bi-directional M values for each of their 100 replicates.

## Results

A total of 627 bp for cyt-*b* and 622 bp for ND4 were sequenced for 181 individuals. Control region sequences of 652 bp were obtained for 143 individuals. Full three-gene concatenated sequences of 1,901 bp were assembled for 141 individuals. These sequences are available in GenBank under accession numbers JN967113-JN967617. Within these 141 individuals, a total of 54 segregating sites were found for a nucleotide diversity (π) of 0.00477, with 34 sites being parsimony informative. A total of 36 haplotypes were present yielding a haplotype diversity of 0.853 (SD = 0.021).

**Figure 2:**
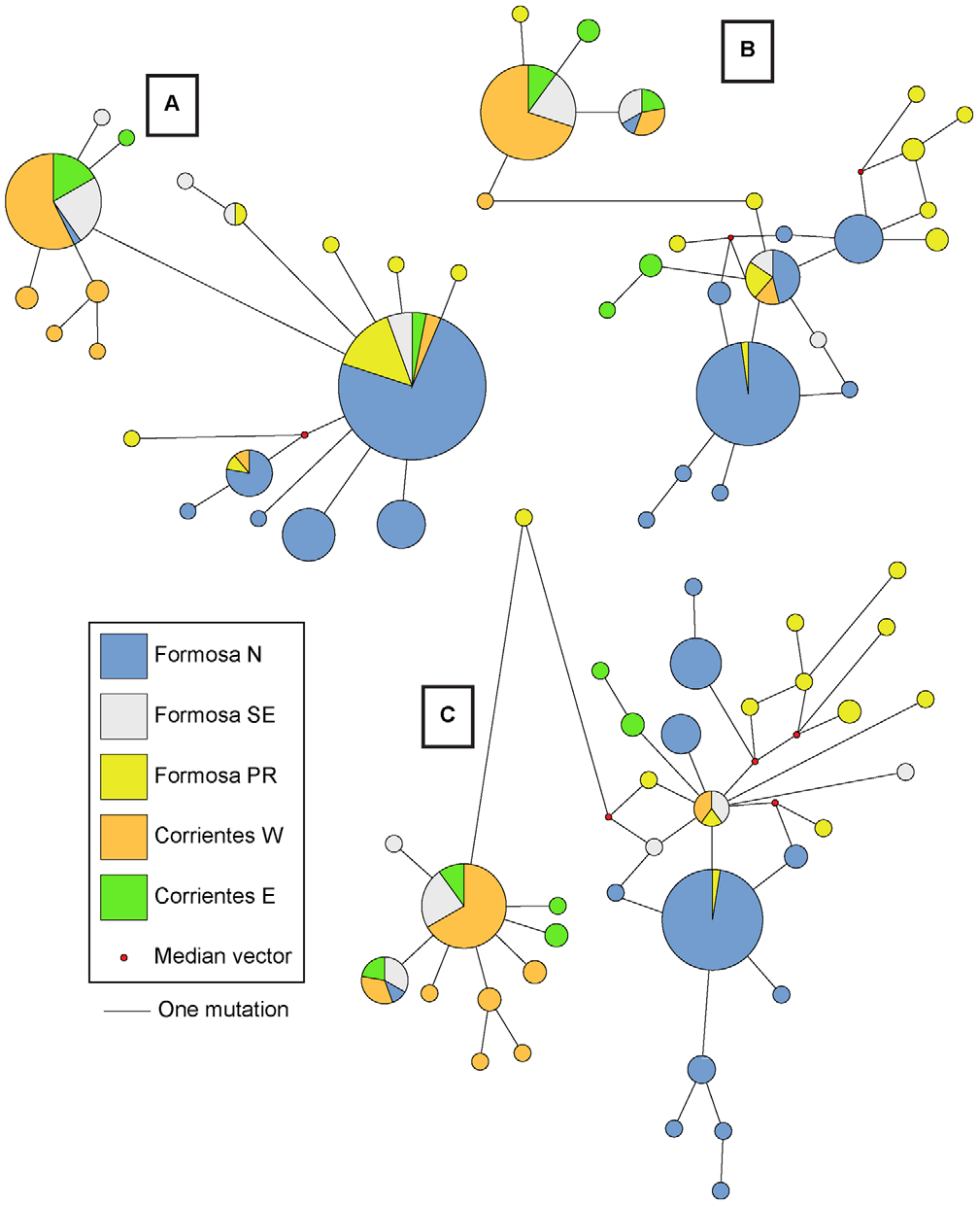
Median-joining haplotype networks. A) cyt-*b* and ND4 concatenated network with 181 individuals (1249 bp). B) control region network representing 143 individuals (652 bp). C) concatenated cyt-*b*, ND4, and control region median-joining network for 141 individuals (1,901 bp). Distances between haplotypes are proportional to number of mutations and are measured from the edge of each circle for all networks. Size of circle indicates relative abundance of haplotype.

Median-joining networks show strong geographic patterns, with control region sequences offering increased resolution over the cyt-*b*/ND4 network, and the three-gene concatenated network showing the clearest overall geographic structuring (Figure 2). All networks show Formosa SE clustering with the two Corrientes populations, and Formosa N increasingly segregating from Formosa PR as more data is added. All putative populations contained unique haplotypes.

Fixation indices showed significant differentiation (p<0.05) between most putative populations (Table 1). Using the three-gene concatenated dataset, all pairwise Fst comparisons were significant except for Formosa SE to Corrientes E (p = 0.28938) and Formosa SE to Corrientes W (p = 0.35016). Pairwise Φ_st_ comparisons were all significant at the 0.05 significance level except for Formosa SE to Corrientes E (p = 0.66667). The exact test of population differentiation [29] showed congruent results, with all pairwise comparisons exhibiting significant differentiation (p<0.05) except for Formosa SE to Corrientes E (p = 0.16095) and Formosa SE to Corrientes W (p = 0.32102).

**Table 1:** Pairwise fixation index results.

Pairwise comparison	Fst	Fst p value	Φst	Φst p value	ETPD p value
**CE to CW**	0.09809	**0.01841**	0.12490	**0.01683**	**0.01134**
**CE to FN**	0.28053	**0.00000**	0.74191	**0.00000**	**0.00000**
**CE to FPR**	0.06003	**0.00020**	0.54024	**0.00010**	**0.01325**
**CE to FSE**	0.01401	0.28938	−0.05158	0.66667	0.16095
**CW to FN**	0.38731	**0.00000**	0.84638	**0.00000**	**0.00000**
**CW to FPR**	0.22630	**0.00100**	0.78497	**0.00000**	**0.00000**
**CW to FSE**	0.00110	0.35016	0.10508	**0.03356**	0.32102
**FN to FPR**	0.19574	**0.00010**	0.23406	**0.00000**	**0.00000**
**FN to FSE**	0.32692	**0.00000**	0.72053	**0.00000**	**0.00000**
**FPR to FSE**	0.11850	**0.00356**	0.52530	**0.00000**	**0.00185**

Tests for IBD were significant (p<0.05) when measuring geographic distance between putative populations along rivers, and not significant (p>0.05) when measuring straight-line distance between putative populations, for both genetic distance measures (Table 2). Our environmental data did not yield significant results in a partial Mantel test for IBED: differences between populations in average monthly precipitation, driest-month average precipitation, whole-year mean temperature, coldest-month mean temperature, and coldest three months mean minimum temperature were not significantly correlated with either measure of genetic distance when controlling for geographical distance (p>0.05) (Table 2).

**Table 2:** Isolation by distance (IBD) and isolation by environmental distance (IBED) results.

	p<	Z	r	r^2^
**Fst–straight-line distance**	0.24913	12.95211	0.294676	8.68E-02
Mean monthly precipitation	0.3348		0.124373	
Driest month mean precipitation	0.78528		−0.32945	
Mean temperature	0.25843		0.24056	
Mean temp. coldest month	0.41454		0.03262	
Mean min. temp. coldest three months	0.40795		0.044684	
**phiST–straight-line distance**	0.10798	95.86122	0.405585	1.64E-01
Mean monthly precipitation	0.55068		−0.10928	
Driest month mean precipitation	0.89269		−0.43677	
Mean temperature	0.20096		0.344395	
Mean temperature coldest month	0.34925		0.20288	
Mean min. temp. coldest three months	0.27414		0.235016	
**Fst–along-river**	0.02481	1513839	0.801239	6.42E-01
Mean monthly precipitation	0.28146		0.185364	
Driest month mean precipitation	0.80767		−0.36982	
Mean temperature	0.26681		0.087927	
Mean temperature coldest month	0.34944		0.057204	
Mean min. temp. coldest three months	0.23417		0.300156	
**phiST–along-river**	0.01674	11172506	0.685424	4.70E-01
Mean monthly precipitation	0.54487		−0.11218	
Driest month mean precipitation	0.8758		−0.391	
Mean temperature	0.2577		0.338462	
Mean temperature coldest month	0.27554		0.332286	
Mean min. temp. coldest three months	0.08304		0.496369	

All three series of MIGRATE runs produced consistent results that indicate asymmetric gene flow in the study area (Table 3). Within the first series 8 of the 10 runs were identical qualitatively and only showed differences in the magnitude of M and *θ*; the remaining 2 runs showed some qualitative differences as well. The second and third series were almost identical qualitatively, with the third series displaying two additional non-zero pairwise M values as a result of the larger initial parameters. Specifically, all three series produced the following agreeing results: positive and relatively large values of gene flow from Formosa PR to Formosa N (with little gene flow in the opposite direction), from Formosa SE to Corrientes E, from Corrientes E to Formosa PR, and from Corrientes W to Formosa SE. The third series also produced positive gene flow to Corrientes W from Formosa PR and from Formosa SE, and smaller gene flow from Formosa SE to Formosa PR. Finally, the two non-identical runs in the first series produced five additional cases of very small gene flow (about 10% of the other values), all of which were single occurrences (frequency of 1).

**Table 3:** Results of migration analysis.

	Corrientes E (CE)			Corrientes W (CW)			Formosa N (FN)			Formosa PR (FPR)			Formosa SE (FSE)		
	s1	s2	s3	s1	s2	s3	s1	s2	s3	s1	s2	s3	s1	s2	s3
**CE**	*θ = 0.012*	*θ = 0.011*	*θ = 0.006*	0.0 (0)	0.0	0.0	0.0 (0)	0.0	0.0	24.6 (1)	0.0	0.0	266.4 (10)	104.1	1690
**CW**	0.0 (0)	0.0	0.0	*θ = 0.010*	*θ = 0.011*	*θ = 0.008*	0.0 (0)	0.0	0.0	0.0 (0)	0.0	65.7	5.2 (1)	0.0	175.4
**FN**	0.0 (0)	0.0	0.0	0.0 (0)	0.0	0.0	*θ = 0.010*	*θ = 0.008*	*θ = 0.012*	87.2 (7)	34.2	1140.0	0.0 (0)	0.0	0.0
**FPR**	53.2 (8)	65.5	315.7	0.0 (0)	0.0	0.0	7.9 (1)	0.0	0.0	*θ = 0.008*	*θ = 0.008*	*θ = 0.010*	5.0 (1)	0.0	31.6
**FSE**	6.1 (1)	0.0	0.0	223.0 (9)	111.5	1090.0	4.1 (1)	0.0	0.0	15.4 (2)	0.0	0.0	*θ = 0.012*	*θ = 0.004*	*θ = 0.011*

Chart is read starting from population in the top row towards the population in the leftmost column. s1: series 1, 10 runs with 10 replicates, number in parenthesis indicates number out of 10 runs that showed positive migration values. s2: series 2, 100 independent replicates s3: series 3, 100 independent replicates with initial M values multiplied by 100 Numbers on diagonal correspond to theta values (2Neµ).

## Discussion

Our analysis revealed clear evidence of spatial structure of yellow anacondas in the study area. Interestingly, as we detail below, such structure cannot be fully explained by simple spatial patterns but rather by a combination of spatial, environmental, and ecological factors.

Most putative population comparisons showed very strong population structuring, which is likely a result of the relative autonomy of the different wetland systems in our study area and also the absence of suitable habitat between populations throughout the wide latitudinal gradient they occupy. Comparisons of genetic structuring with other large semiaquatic snakes are difficult due to lack of published genetic studies in large snakes. Lower levels of population structuring were found in studies of the closely-related Argentine boa constrictor, *Boa constrictor occidentalis* [39], [40], where the authors also found evidence for sex-biased dispersal. This is an interesting comparison as the boa constrictor prefers dry forests and is not limited to riverine habitat, therefore allowing us to evaluate opposite landscape and environmental constraints to dispersal [40], [41]. In that case, the authors found that loss of landscape connectivity in the form of degraded habitat between suitable forest patches led to lower levels of gene flow between populations [40]. Though it is smaller than *Eunectes notaeus*, the northern water snake (*Nerodia sipedon sipedon*) from Ontario, Canada has shown evidence of population structure between populations much closer together than those in our analysis (less than 2 km apart), and dry areas were found to greatly reduce capacity for dispersal for this aquatic snake [42]. In contrast, Meister et. al [43] found no evidence of genetic structuring in wetland-associated populations of grass snakes (*Natrix natrix*) over a 90 km^2^ area of habitat highly fragmented by agriculture. Dispersal capabilities are slightly better understood for large snakes, as there have been several ecological studies done. For instance, Rivas et. al discovered that large female green anacondas (*Eunectes murinus*) move very little, and large individuals typically move less than 20–30 m for several weeks after feeding or during pregnancy [44].

The reliability of our analysis and observed patterns stem from the power of the data we analyzed. It is often suggested that adding individuals or genetic markers results in increased resolution of performed analyses (such as [45] and [46]), though few report this finding empirically (as in [47]). This idea is supported here, as increased sample sizes and additional genetic markers clearly improved the resolution over an earlier study on the same system [1]. This effect was also evident within the median-joining networks used in this study, as increased coverage of the mitochondrial genome, especially the inclusion of control region sequences, led to increased spatial resolution (Figure 2). This agrees with the concept that the mitochondrial control region diverges faster and provides greater resolution in phylogenetic and population genetic analyses of closely-related individuals than do other regions of the mitochondrial genome [45], [48].

In parallel to the evident power of our analyses and resolution in our data, it is important to highlight the inherent limitations of genetic analyses focusing on matrilineal markers. Yellow anacondas exhibit a high degree of sexual size dimorphism, with females attaining weights approximately twice that of males [10]. This is relevant to our analysis as larger individuals (notably females) may not disperse as readily as smaller, more mobile individuals. In fact, direct evidence of males moving more than females while searching for mating partners has been observed in the congeneric green anaconda (*Eunectes murinus*) [44]. The limited mobility of large females is important, as they have the highest fecundity [10], [49], and the mitochondrial DNA used in this analysis is maternally inherited. This indicates that the individuals of the species with the lowest mobility contribute the most to the populations and to our analysis. Because of this, and also because sex-biased dispersal has been observed in other snakes including *Boa constrictor* [39], [50], [51], additional analyses may be needed to fully understand demographic dispersal in this species. Addition of nuclear markers or Y-chromosome data could clarify the role of male dispersal in this system, and help us understand the full degree of gene flow occurring. If analysis of nuclear or Y-chromosome data showed different results than those found in this study, then that could be evidence for sex-biased dispersal [52].

Although anacondas appear to be using rivers to disperse, they do not use them as channels to swim with the current. However, *Eunectes notaeus* requires habitat that is associated with rivers, like floodplains, and floating vegetation may influence their directionality of movement. Snakes move along the marshes abutting the rivers in both directions: upstream and downstream. As such, traditional methods of testing for IBD using straight-line distance between populations are inappropriate. By instead measuring linear distance along rivers we can better approximate the distance snakes must travel to reach other populations. Significant support for IBD using along-river distance, together with nonsignificant tests for IBD using Euclidean distance, indicates that rivers and their associated floodplains are important in the dispersal of this species. The environmental variables used in this study did not prove useful in predicting genetic distance between populations, as more complex and finer-scaled environmental variables are likely needed to accurately predict environmental isolation in this system. For instance, precipitation within the study area will be less important than presence or absence of riverine or flooded habitat. Variables and analyses that can give a better representation of suitable habitat will likely be more relevant in uncovering potential environmental isolation. Several options exist, such as predicting flooding with a digital elevation model and inundation simulation [53], [54] or measuring “wetness” using NASA Landsat data and a Tasseled-Cap transformation [55]. Alternatively, more complex environmental niche modeling might be possible with suitable data and software such as Maxent [56], [57], where the resulting fine-scale knowledge of suitable and unsuitable habitat for *Eunectes notaeus* would allow for additional approaches such as least-cost path [5], [58] or circuit theory [59]–[61] analyses.

Confirming that riverine habitat is important to yellow anaconda dispersal allows us to focus on specific aspects of rivers that might be important to dispersal. Our gene flow analysis shows clear evidence of asymmetric gene flow, which indicates that the barriers to dispersal (or historical colonization opportunities) are also asymmetric. Rivers are inherently directional in their flow, and this very likely contributes to the patterns of migration found here. Directional gene flow has previously been found in other snakes [50], [62], [63], suggesting that this could be a rather frequent pattern, especially in species whose habitat preference for riverine habitats is strong. For example, Dubey et al. [50] found support for asymmetric gene flow between several populations of Australian slaty-grey snake (*Stegonotus cucullatus*) from the riparian zone around the Adelaide River in the Northern Territory of Australia. Investigating more species exclusive to riverine habitat would help to reveal to what degree rivers drive asymmetric migration and gene flow.

Putting our analyses in light of the historical geomorphology of our study area and the natural history of our species allows a better understanding of the issues we sought to evaluate. Our MIGRATE analysis shows that gene flow occurs from Formosa PR to Formosa N, but not in the opposite direction. The strong directional gene flow likely reflects the colonization events of Formosa N. Specifically, it could be the result of the highly dynamic hydrological processes that established the La Estrella marshes several decades ago [10], [16]. Until the 1960s the La Estrella marshes drained through rivers located in the neighboring country of Paraguay, when suddenly this marsh changed its position and activated most of the small creeks and rivers of the Argentine side located at what we call Formosa N. La Estrella marshlands waters currently flow from northwest to southeast through different wetlands and creeks to finally end in the Paraguay River. In this sense, the reactivation of this “connection” is relatively recent, estimated to have occurred less than 50 years, and our data reflects this colonization event from Formosa PR to Formosa N. The historical signature of colonization remains visible in the migration analysis, even with weak modern-day dispersal opposing it.

Gene flow was detected from Formosa PR to Corrientes W, and not in the reverse direction. The directionality and strong flow of the Paraguay and Paraná rivers probably aids in the dispersal of vegetation and anacondas in a southerly direction, though only a small degree of gene flow was detected, and was only found in the MIGRATE analysis with extremely high starting M values. This may indicate that the strength of river flow is not as important for dispersal as other factors such as presence of suitable habitat in the areas surrounding rivers (i.e. floodplains). A low degree of gene flow between populations is supported by strong and highly significant values for fixation indices between Formosa PR and Corrientes W. The population at Formosa PR is situated on the Paraguay River downstream of wetlands in Paraguay and Brazil, where yellow anacondas are also found. Therefore there may be some unaccounted for migration of unsampled populations into our system, also known as a ghost population [64], [65]. If migration is occurring from Brazil and Paraguay to Argentina, any influence would be most obvious in the Formosa PR population, and could contribute to its significant differentiation between it and all other populations in our system.

Migration from Corrientes E individuals to Formosa PR may also dilute the effect of Formosa PR to Corrientes W migration. Strong and consistent gene flow was discovered from Corrientes E to Formosa PR, but little to no migration was found in the opposite direction. Corrientes E swamps and marshlands are located along the ancient alluvial cone of the Paraná River, and its waters slowly flow from east to west. This combined with continuous favorable habitat supports the possibility of directional migration between these two populations.

The patterns of gene flow between Formosa SE, Corrientes W, and Corrientes E are less intuitive. These three populations show that movement in this system is bidirectional but asymmetric. Migration was found from Formosa SE to Corrientes W and Corrientes E, and also from Corrientes W to Formosa SE. This appears to indicate strong upstream dispersal from Formosa SE to Corrientes E along the Paraná River and its ancient alluvial valley now covered with multiple swamp systems (with a much lower rate of gene flow in the opposite direction), and also upstream dispersal from Corrientes W to Formosa SE along the Paraná and Paraguay rivers and through the Paraguay River tributary streams. Since these populations are found relatively far away from their closest river, the observed patterns of gene flow could reflect a demographic history of connectivity by more extensive suitable habitat, rather than current gene flow. Small amounts of gene flow were also found from Formosa SE to Corrientes W, which follows the directionality of the Paraguay and Paraná rivers. Fixation indices support the presence of gene flow and lack of differentiation between these populations, as Fst values were nonsignificant for Formosa SE/Corrientes E and Formosa SE/Corrientes W.

Genetic analysis helps us delineate management units and augments our understanding of demographic processes, which is particularly valuable in species such as aquatic organisms, which are notoriously difficult to observe. *Eunectes notaeus* is currently being harvested under a sustainable management plan in a single wetland located within Formosa N [2] (Micucci, Waller, Draque, Barros, and Lerea (2011) Programa Curiyú – ampaña 2010. Fundación Biodiversidad, unpublished report). Scientifically-based management schemes are appropriate steps to achieve the sustainable use of a species against historical patterns of indiscriminate harvesting, and reducing uncertainty through science is a major goal under these programs [10], [66], [67]. Based on the evidence we gathered in our study, if new management plans are established we suggest they should consider the putative populations Formosa N, Formosa PR, Corrientes W, and combined Formosa SE and Corrientes E to be distinct management units. More generally, since the species exhibits significant genetic structuring in relation to different hydrological systems, the conservation planning and sustainable use of this species should consider clearly delimited wetland systems as potential management units when no other information is available. The strong population structure and directional migration found with these genetic markers suggest that some populations, if threatened, may not be easily “rescued”by distant populations, confirming that the harvesting of these populations should only be allowed under scientifically-sound policies. Further analysis using different genetic markers to test for sex-biased dispersal and more complex habitat modeling may prove beneficial to understanding the spatial ecology of these animals.
