# Correction: Population Structure and Gene Flow of the Yellow Anaconda (*Eunectes notaeus*) in Northern Argentina

**DOI:** 10.1371/annotation/06482a0a-b49c-4feb-9aa8-d3c8f72e90fb

**Published:** 2012-09-05

**Authors:** Evan McCartney-Melstad, Tomás Waller, Patricio A. Micucci, Mariano Barros, Juan Draque, George Amato, Martin Mendez

There was an error in the affiliations for author Martin Mendez. The author should be affiliated with 2. Sackler Institute for Comparative Genomics, American Museum of Natural History, New York, New York, United States of America, 4. Wildlife Conservation Society, Bronx, New York, United States of America, as well as being corresponding author. 

